# Lin28 affects the proliferation and osteogenic differentiation of human dental pulp stem cells by directly inhibiting let-7b maturation

**DOI:** 10.1038/s41405-024-00194-8

**Published:** 2024-03-05

**Authors:** Liu Yan, Jing Sun, Yushan Wang, Xinxin Liu, Jiayi Hu, Mengxin Sun, Xi Suo, Rongquan Duan, Changyong Yuan

**Affiliations:** 1 School of Stomatology, Xuzhou Medical University, No.209 Tongshan Road, Xuzhou, 221004 Jiangsu China; 2 Affiliated Stomatological Hospital of Xuzhou Medical University, No.130 Huaihai West Road, Xuzhou, 221000 Jiangsu China; 3 School and Hospital of Stomatology, Cheeloo College of Medicine, Shandong University, Jinan, 250012 Shandong China

**Keywords:** Pulpitis, Pulp conservation

## Abstract

**Objective:**

Activation of Lin28 gene under certain conditions promotes tissue damage repair. However, it remains unknown whether conditional expression of Lin28 facilitates the recovery of damaged pulp tissue. In the study, we focus on exploring the effects and possible regulatory mechanisms of Lin28 on the proliferation and differentiation of human dental pulp stem cells (hDPSCs).

**Materials and methods:**

We adopted techniques such as the ethynyl-2ʹ-deoxyuridine (EdU) incorporation assay, RNA-protein immunoprecipitation (RIP) analysis, and luciferase assays to study the regulation of hDPSCs by Lin28. Furthermore, gain-of-function and loss-of-function analyses were also used in explored factors regulating hDPSCs activation.

**Results:**

The results show that Lin28 inhibited osteogenic differentiation by directly targets pre-let-7b. Through bioinformatics sequencing and dual luciferase experiments we learned that let-7b directly targets the IGF2BP2 3’UTR. Silencing of IGF2BP2 showed a similar biological effect as overexpression of let-7b. Overexpression of IGF2BP2 counteracted the differentiation-promoting effects produced by let-7b overexpression.

**Discussion/conclusions:**

In conclusion, the RNA-binding protein Lin28 regulates osteogenic differentiation of hDPSCs by inhibiting let-7 miRNA maturation. And mature let-7b directly regulated the expression of IGF2BP2 by targeting the 3’UTR region of IGF2BP2 mRNA thus further inhibiting the differentiation of hDPSCs.

## Introduction

Dental caries and other dental diseases usually lead to non-vital tooth or even teeth loss, if left untreated. Clinical therapy for these diseases can be unpredictable, often resulting in the need for endodontic therapy. Moreover, jaw defects are an important issue in maxillofacial surgery. With the rapid development of tissue engineering and stem cell biology, the use of stem cells to promote bone regeneration has become more popular.

Depending on the source, stem cells can be divided into embryonic and adult stem cells [1]. Cataldi et al. found that there is a type of adult stem cell in human dental pulp tissue with transdermal differentiation ability [2–5], whilst retaining the plasticity of stem cells [6]. Autogenous (from the third molar, orthodontically extracted tooth) human dental pulp stem cells have become a new research hotspot in dental and periodontal medicine for the treatment of dental pulp and periodontal bone diseases and restoration of the physiological function of teeth. Consequently, stimulating natural odontogenesis from an improved stem cell-based therapy is expected [7]. Dental stem cells, such as human dental pulp stem cells (hDPSCs) derived from human dental pulp, can differentiate into odontoblastic lineages in vitro [8]. In addition, transplantation into immunocompromised mice revealed that hDPSCs have the potential to generate ectopic dentin-pulp-like complexes [9]. In addition, hDPSCs are abundant, can be collected painlessly with minimal invasion, have low immunogenicity and high odonto/osteogenic differentiation ability, and do not pose major ethical considerations [10]. Numerous growth factors have also been reported to induce the differentiation and mineralization of dental pulp cells [11, 12]; however, their precise mechanisms remain unclear [13].

Lin28 was initially identified as a heterochronic gene in *Caenorhabditis elegans* that regulates developmental timing. It is a highly conserved RNA-binding protein expressed during embryogenesis that regulates several important cellular functions associated with development, glucose metabolism, and pluripotency. Lin28 expression halts at birth, and its reactivation leads to an elevation of the regenerative capacity of various tissues in young adult mice, such as hair regrowth by promoting anagen in hair follicles, and the regrowth of cartilage, bone, and mesenchyme following ear and digit injuries [14]. Lin28 binds directly to numerous messenger RNAs (mRNAs) that regulate metabolic processes and cell growth [15, 16]. Our previous study showed that Lin28 was expressed and distributed during tooth development and may play a positive role in ameloblast and odontoblast differentiation, matrix secretion, and mineralization of enamel and dentin [17, 18]. Our previous in vitro study demonstrated that Lin28 promotes dental pulp cell proliferation via the let-7a/ insulin-like growth factor 2 mRNA-binding protein 2 (IGF2BP2) pathway [13]. Thus, Lin28 directly helps control the metabolic state of cells, which is crucial for tissue generation.

Previously, the role of IGF2BP2 was thought to be limited to genes involved in type 2 diabetes [19]. IGF2BP2 is an n6-methyladenosine (m6A) reader that can communicate with various micro-RNAs (miRNAs), mRNAs, and long non-coding RNAs (lncRNAs) [20]. It also affects the transcription of many genes and regulates pathways in metabolic diseases, such as fatty liver, cardiovascular diseases, and tumors, thereby affecting cell metabolism [21, 22]. It has been reported that IGF2BP2 plays a vital role in a wide range of physiological processes, such as body growth, embryonic development, and metabolism. Its regulatory role in differentiating neural precursor cells into neurons or glial cells is closely related to its expression during early embryonic development, which decreases after birth [23]. However, the effect of Lin28 or IGF2BP2 on the odontogenic differentiation of hDPSCs remains unknown.

This study aimed to elucidate the function and mechanism of Lin28, let-7b, and IGF2BP2 in the osteo/odontoblastic differentiation of hDPSCs, and evaluate the possible function of let-7b during this process. Our results illustrate the effects of Lin28/IGF2BP2 on reducing the terminal odontoblastic differentiation of hDPSCs by interacting with let-7b.

## Methods

### Cell isolation and culture

Intact caries-free teeth (third molars or premolars) werecollected from patients at the Stomatology Hospital of Xuzhou Medical University. This study was approved by the Institutional Research Ethics Committee of Xuzhou Medical University (2022-KY-013). Briefly, hDPSCs were isolated and cultured as previously reported [13, 24]. Cells obtained between passages two and five were used in subsequent experiments.

### Dental pulp stem cell characterization

To determine the presence of a typical mesenchymal stem cell immunophenotype, hDPSCs were collected and incubated for 30 min at 4 °C in the dark with the following monoclonal antibodies: anti-Cluster of Differentiation (CD)90-Peridinin chlorophyll protein (PERCP), anti-D73-fluorescein isothiocyanate (FITC), anti-CD45- Allophycocyanin (APC)105 (BD Biosciences, San Jose, CA, USA), and anti-STRO-1- phycoerythrin (PE) (Santa Cruz Biotechnology, Santa Cruz, CA, USA). The stained cells were washed and resuspended in 0.01 M phosphate buffer solution (PBS) with 4% paraformaldehyde. The surface molecules of all samples were analyzed using a BD FACSCalibur (BD Biosciences). Immunohistochemical staining for vimentin and keratin (Abcam, USA) was also carried out to verify the origin of the cell lines. To confirm their pluripotency, hDPSCs were seeded in 12-well plates and cultured in an osteogenic and adipogenic medium for 14 d. The osteogenic medium contained α-MEM supplemented with 10% FBS, 100 µ/mL penicillin–streptomycin (HyClone), 10 nM dexamethasone, 1 M sodium β‐glycerophosphate, and 5 mg/mL L‐ascorbic acid (Sigma, St. Louis, MO, USA). Alizarin red, alkaline phosphatase (ALP) staining and oil red staining were used to characterize the osteogenic and lipogenic differentiation capacity of dental pulp stem cells, respectively [25]. Data were from three independent experiments.

### Lentiviral and transient transfection of hDPSCs

Lentiviral (LV) packages expressing Lin28 and IGF2BP2 were constructed by GenePharma (Shanghai, China) and named LV-Lin28 (LV-Lin28-green fluorescent protein (GFP)-Puro) and LV-IGF2BP2 (LV-IGF2BP2-GFP-Puro), respectively. A scrambled LV vector, small hairpin (sh)-IGF2BP2 (LV-IGF2BP2-GFP-Puro), was used as a control. Upon reaching 20–30% confluency, the cells were treated with LV particles at a multiplicity of infection of 200. At 72 h after transfection, transfection efficiency was observed under a fluorescence microscope (Teneo VS; Field Electron and Ion Company (FEI), USA). Quantitative reverse transcription-polymerase chain reaction (qRT-PCR) and western blotting analyses were performed to evaluate the expression of Lin28, let-7b, and IGF2BP2 mRNA and protein, respectively. Following this method, hDPSCs were transfected with let-7b mimics, agomiRs, inhibitor particles (GenePharma), and scrambled miRNA (as a negative control) to regulate let-7b levels transiently, as well as IGF2BP2 small interfering RNA (siRNA) and shRNA (GenePharma) to knock down IGF2BP2 gene expression. The oligonucleotide sequences of the target genes in this study are presented in Table 1. Data were from three independent experiments.

**Table 1:** Sequence of various primers and oligonucleotides used in this study.

Name	Sequence (5ʹ-3ʹ)
Lin28 F	GCACCAGAGTAAGCTGCACA
Lin28 R	GAATAGCCCCCACCCATTGT
β-actin F	AGCGAGCATCCCCCAAAGTT
β-actin R	GGGCACGAAGGCTCATCATT
let-7b-5p sense	UGAGGUAGUAGGUUGUGUGGUU
let-7b-5p antisense	AACCCACACAACCUACUACCUCA
mimics ctrl sense	UUCUCCGAACGUGUCACGUTT
mimics ctrl antisense	ACGUGACACGUUCGGAGAATT
let-7b-5p inhibitor	AACCACACAACCUACUACCUCA
inhibitor ctrl	CAGUACUUUUGUGUAGUACAA
let-7b agomir sense	UGAGGUAGUAGGUUGUGUGGUU
let-7b agomir antisense	CCACACAACCUACUACCUCAUU
let-7b agomir ctrl sense	UUCUCCGAACGUGUCACGUTT
let-7b agomir ctrl antisense	ACGUGACACGUUCGGAGAATT
let-7b RT	GTCGTATCCAGTGCGTGTCGTGGAGTCGGCAATTGCACTGGATACGACCAGGGAA
let-7b F	ATCCAGTGCGTGTCGTG
let-7b R	TGCTTGAGGTAGTAGGTTG
U6 RT	CGCTTCACGAATTTGCGTGTCAT
U6 F	GCTTCGGCAGCACATATACTAAAAT
U6 R	CGCTTCACGAATTTGCGTGTCAT
IGF2BP2 F	CTGGCCGTGTTCCGGGAGAA
IGF2BP2 R	TTCCTGTTGGCAGGGAGTCCTGG
IGF2BP2-WT-sense	CGAAAAACACACAGAAGAAGCTACCTCAGGTGTTTTTACCTCAGCACCTTGCTCTTGTGTTC
IGF2BP2-WT-antisense	TCGAGAACACAAGAGCAAGGTGCTGAGGTAAAAACACCTGAGGTAGCTTCTTCTGTGTGTTTTTCGAGCT
IGF2BP2-Mut1-sense	CGAAAAACACTGTGAAGAAGGATGGAGAGGTGTTTTTACCTCAGCACCTTGCTCTTGTGTTC
IGF2BP2-Mut1-antisense	TCGAGAACACAAGAGCAAGGTGCTGAGGTAAAAACACCTCTCCATCCTTCTTCACAGTGTTTTTCGAGCT
IGF2BP2-Mut2-sense	CGAAAAACACACAGAAGAAGCTACCTCAGGTGTTTTTATGGAGGCACCTTGCTCTTGTGTTC
IGF2BP2-Mut2-antisense	TCGAGAACACAAGAGCAAGGTGCCTCCATAAAAACACCTGAGGTAGCTTCTTCTGTGTGTTTTTCGAGCT
siRNA ctrl sense	UUCUCCGAACGUGUCACGUTT
siRNA ctrl antisense	ACGUGACACGUUCGGAGAATT
si-IGF2BP2-1 sense	CAGUUUGAGAACUACUCCUTT
si-IGF2BP2-1 antisense	AGGAGUAGUUCUCAAACUGTT
si-IGF2BP2-2 sense	GUGAAUCUCUUCAUCCCAATT
si-IGF2BP2-2 antisense	UUGGGAUGAAGAGAUUCACTT

### Alizarin red staining

Transfected cells (2.5 × 10^5^ cells/well) seeded in six-well plates were allowed to undergo odontogenic differentiation for 14 and 21 days. The differentiation medium was replaced every other day. After fixing with 4% paraformaldehyde for 30 min, the cells were washed with double-distilled water and incubated with 1% Alizarin red reagent (Sigma) for 40 min. Images were captured using a microscope (Teneo VS; FEI). Data were from three independent experiments.

### Alkaline phosphatase staining

Transfected cells (5 × 10^4^ cells/well) were seeded into 24-well plates (Corning) and subjected to odontogenic differentiation for 14 d. For ALP staining, the cells were fixed in 4% paraformaldehyde for 30 min and stained using a 5-bromo-4-chloro-3-indolyl phosphate (BCIP)/nitro blue tetrazolium (NBT) ALP Color Development Kit (Beyotime, Shanghai, China) according to the manufacturer’s protocol. Data were from three independent experiments.

### Cell counting kit-8 (CCK-8) assay

The effects of the different treatments on the proliferation of hDPSCs were detected using a CCK-8 kit (Vicmed, Jiangsu, China). Briefly, 5 × 10^3^ hDPSCs were seeded per well of a 96-well plate (Corning). At 24, 48, and 72 h after seeding, a 10-µL aliquot of the CCK-8 reagent and 100 μL of fresh culture medium were simultaneously added to each well, and the plate was incubated for another two hours. Finally, the absorbance of each well at 450 nm was measured using a microculture plate reader (FLUOstar OPTIMA, BMG, Germany). At least five replicate wells were assigned to each treatment group. Data were from three independent experiments.

### Ethynyl-2’-deoxyuridine (EdU) incorporation assay

In order to evaluate proliferation of hDPSCs, the EdU incorporation assay was performed using an EdU Apollo DNA in vitro kit (RiboBio Cell Cycle Analysis, Guangzhou, China), according to the instructions. The methodology can be referred to our previous study [13].

### qRT‐PCR

Total RNA was isolated from hDPSCs using TRIzol reagent (Invitrogen) and reverse-transcribed into complementary DNA (cDNA) using a PrimeScript RT Master Mix kit (TaKaRa, Dalian, China). qRT-PCR was performed using an SYBR Green PCR Master Mix Plus (Takara) in a 20 μL reaction mixture using a flexible test chamber (FTC)-3000 System (Funglyn Biotech Inc., Toronto, Canada). All reactions were performed in triplicate, and the transcription level of each gene was standardized to that of β-actin. Relative gene expression levels were calculated using the 2^−ΔΔCt^ method. The sequences of the primers used in this study are listed in Table 1. Data were from three independent experiments.

### Western blotting analysis

Cells were lysed in ice-cold radioimmunoprecipitation assay buffer (Beyotime) containing phenylmethylsulfonyl fluoride (PMSF) (Beyotime Biotechnology, Haimen, China). The membranes were incubated with primary antibodies overnight, and then incubated with horseradish peroxidase (HRP)-conjugated anti-rabbit (1:5000, Proteintech, Wuhan, Hubei, China) or anti-mouse (1:10,000, Proteintech) secondary antibodies. Band densities were quantified and normalized to β-actin using ImageJ 1.8.0. The following primary antibodies were used: anti-Lin28 (1:1000, Cell Signaling Technology), anti-IGF2BP2 (1:1000, Cell Signaling Technology). Data were from three independent experiments.

### RNA-protein immunoprecipitation (RIP)

To understand the mechanism by which Lin28 regulates the proliferation and osteogenic differentiation of hDPSCs, we performed RIP-Chip analysis. RNAs immunoprecipitated with immunoglobulin G (IgG) were used as a control. A previous study [26] reported that Lin28 binds to more than a thousand transcripts and regulates the translation of these mRNAs in human embryonic stem cells. To analyze the interaction between individual proteins and RNA, we performed RIP according to the manufacturer’s instructions (Millipore, Billerica, MA, USA). The methodology can be referred to our previous study [13].

### Co-immunoprecipitation (co-IP)

A cell lysis buffer containing NP-40 (with a protease inhibitor) was added to the cells, which were lysed on ice for 30 min. The resulting cell lysate was then incubated at 4 °C, and 5 µg anti-Lin28 antibodies were added. Next, protein A/G-mixed agarose beads were added to the cell lysate and incubated overnight to couple the antibodies to the protein A/agarose beads. Following the immunoprecipitation reaction, a 2× sodium dodecyl sulfate loading buffer was added to the cell lysates and boiled for five minutes. Finally, western blotting was performed to determine the relationship between Lin28 and IGF2BP2. Data were from three independent experiments.

### Luciferase reporter assay

First, a set of mRNAs targeted by let-7b was identified, and the let-7b-5p binding sites to IGF2BP2 were further predicted using the MicroRNA Target Prediction Database (miRDB), TargetScan, and microRNA.org bioinformatic analysis website. The 3ʹ-untranslated region (UTR) of IGF2BP2, containing let-7b binding sites at positions 1304–1311 and positions 1320–1326, was cloned downstream of the luciferase reporter in the pmirGLO Dual-Luciferase miRNA Target Expression Vector (Promega, Madison, WI, USA) between the *Streptomyces achromogenes* (*Sac*)I and *Xanthomonas vasicola* (*Xho*)I sites. The methodology can be referred to our previous study [13]. All experiments were performed in triplicate for further statistical analysis. Data were from three independent experiments.

### Statistical analysis

All quantitative data were collected from at least three independent experiments and expressed as the mean and standard deviation. Differences between the two groups were analyzed using Student’s *t* test, and to determine whether the differences between several groups were significant, a one-way analysis of variance was performed. All statistical analyses were performed using Statistical Product and Service Solutions (SPSS) 25.0 software. *P* < 0.05 were considered statistically significant. All analyzed data were visualized using GraphPad Prism 9.0 software.

## Results

### Culture and identification of hDPSCs

**Fig. 1:**
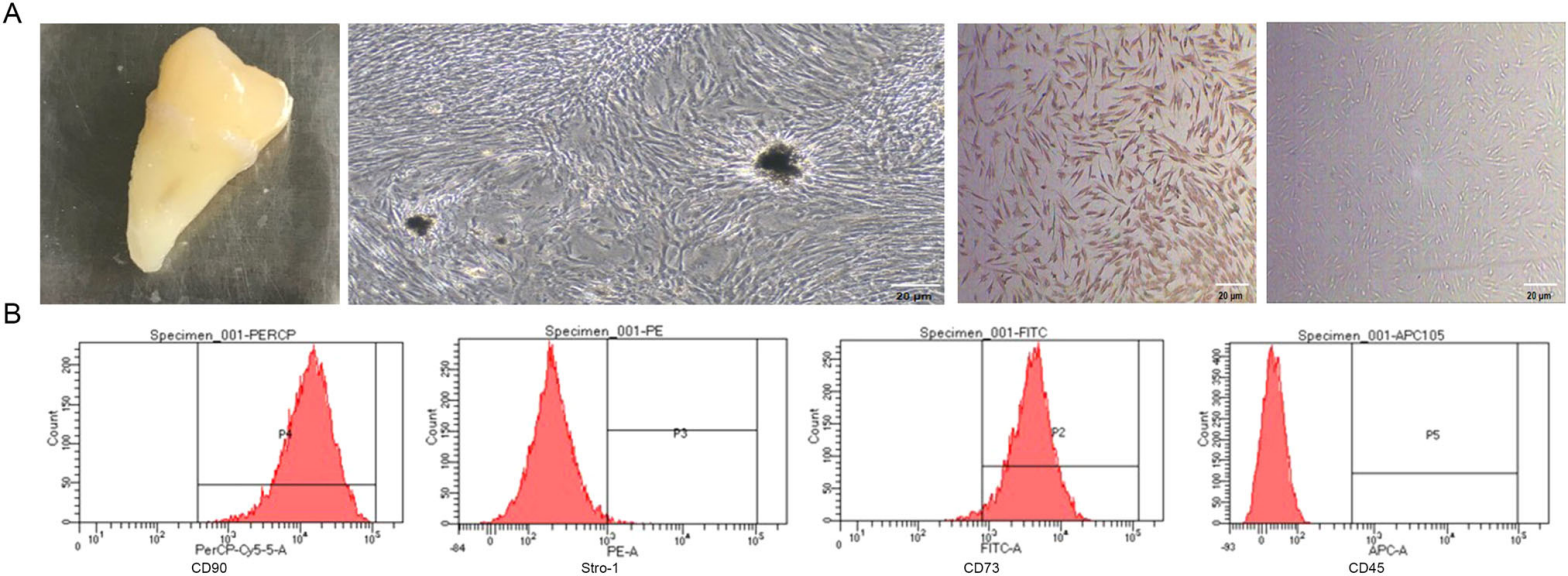
Identification of hDPSCs. **A** Migration of primary dental pulp cells from pulp tissue. Vimentin and keratin identify cells derived from the mesoderm. **B** Flow cytometry detection of stem cells indicating antigens, such as CD90, Stro-1, CD73, CD45. Scar bar = 20 μm.

After one week of culture, long spindle-shaped hDPSCs emerged from the tissue blocks, grew in clusters, and were arranged into tight masses. The spindle-shaped cells scattered around the masses were collected and subjected to flow cytometry for antigen detection (Fig. 1A). Concurrently, immunohistochemical staining showed that vimentin was expressed as brown particles in the cytoplasm, showing strong positive expression and confirming the mesenchymal origin of the cells. The expression of vimentin and keratin was positive and negative, respectively (Fig. 1A), revealing that the hDPSCs were derived from the mesoderm and differentiated from the mesenchyme. The results showed that the positivity rates of the hDPSCs for the surface markers CD90 and CD73 were 99.9% and 97.3%, respectively (Fig. 1B). Additionally, the positivity rate for the early mesenchymal stem cell marker STRO-1 was 1.3%. In contrast, CD45 was not expressed, indicating that these cells had the characteristics of mesenchymal stem cells.

### Lin28 promotes the proliferation and inhibits the differentiation of hDPSCs

**Fig. 2:**
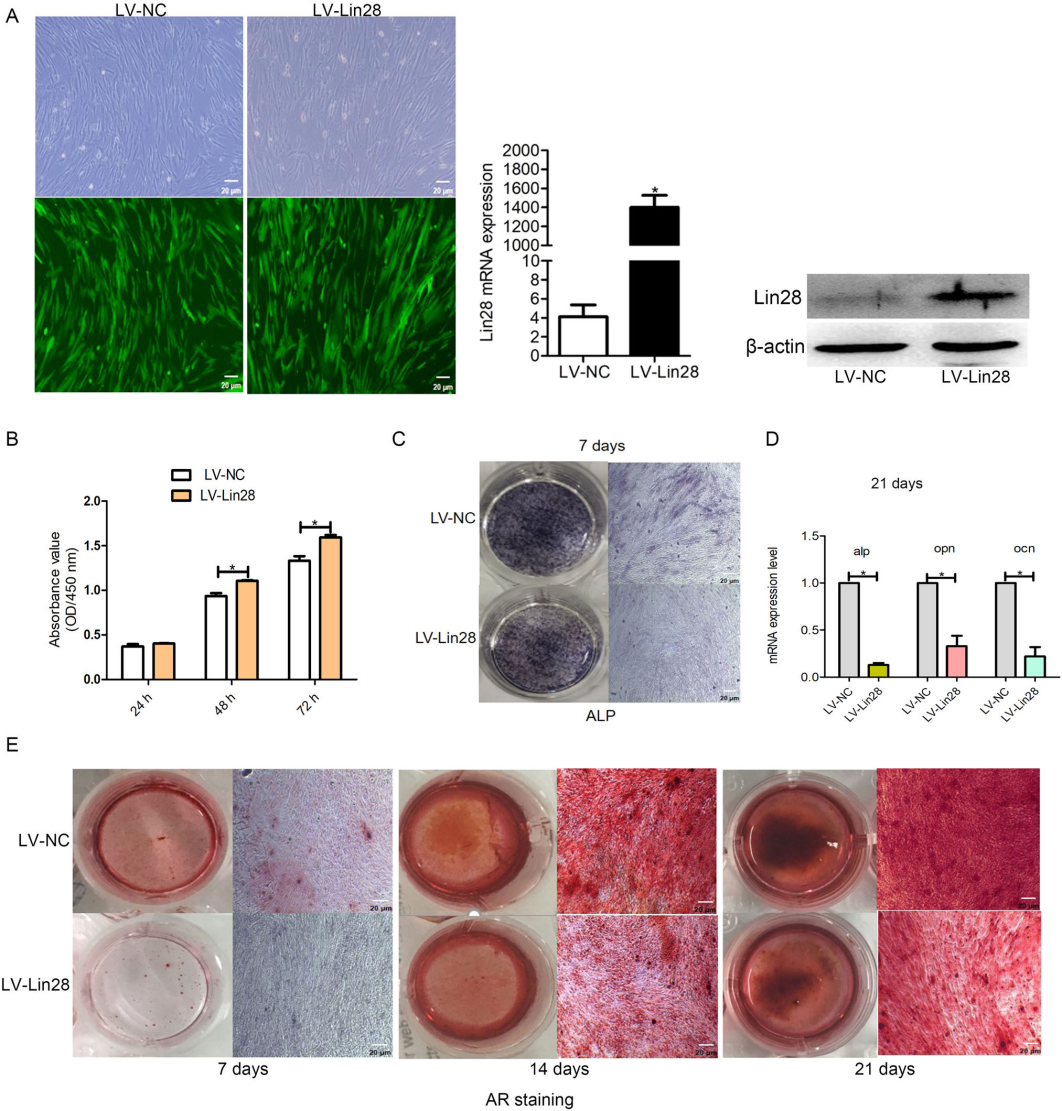
Evaluation of proliferation and differentiation ability of hDPSCs overexpressing Lin28. **A** Detection of Lin28 transfection efficiency in hDPSCs. **B** Detection of proliferative activity of hDPSCs after transfection with Lin28. **C**–**E** ALP, PCR and alizarin red staining were used to detect osteogenic differentiation of hDPSCs after transfection with Lin28, respectively. Scar bar = 20 μm.

To assess the function of Lin28 in vitro, cells were transfected with LV particles, and their total Lin28 mRNA and protein levels were significantly higher than those in scrambled-miRNA-transfected cells (*P* < 0.05; Fig. 2A). The CCK-8 assay revealed that hDPSC proliferation significantly improved over time (*P* < 0.05; Fig. 2B). In addition, the hDPSCs in each group were stained with Alizarin red and ALP staining solution to examine their osteogenic ability, and an inverse relationship between the differentiation capacity of hDPSCs and Lin28 levels was observed (Fig. 2C, E).

Following the above results, qRT-PCR verified that the relative mRNA expression of *ALP*, *OPN*, and osteocalcin (*OCN*) were dramatically improved compared to those in the sham control (*P* < 0.05; Fig. 2D). These data confirmed that cell proliferation was positively correlated, and differentiation ability was negatively correlated with Lin28 levels.

### Let-7b inhibits the proliferation and promotes the differentiation of hDPSCs

**Fig. 3:**
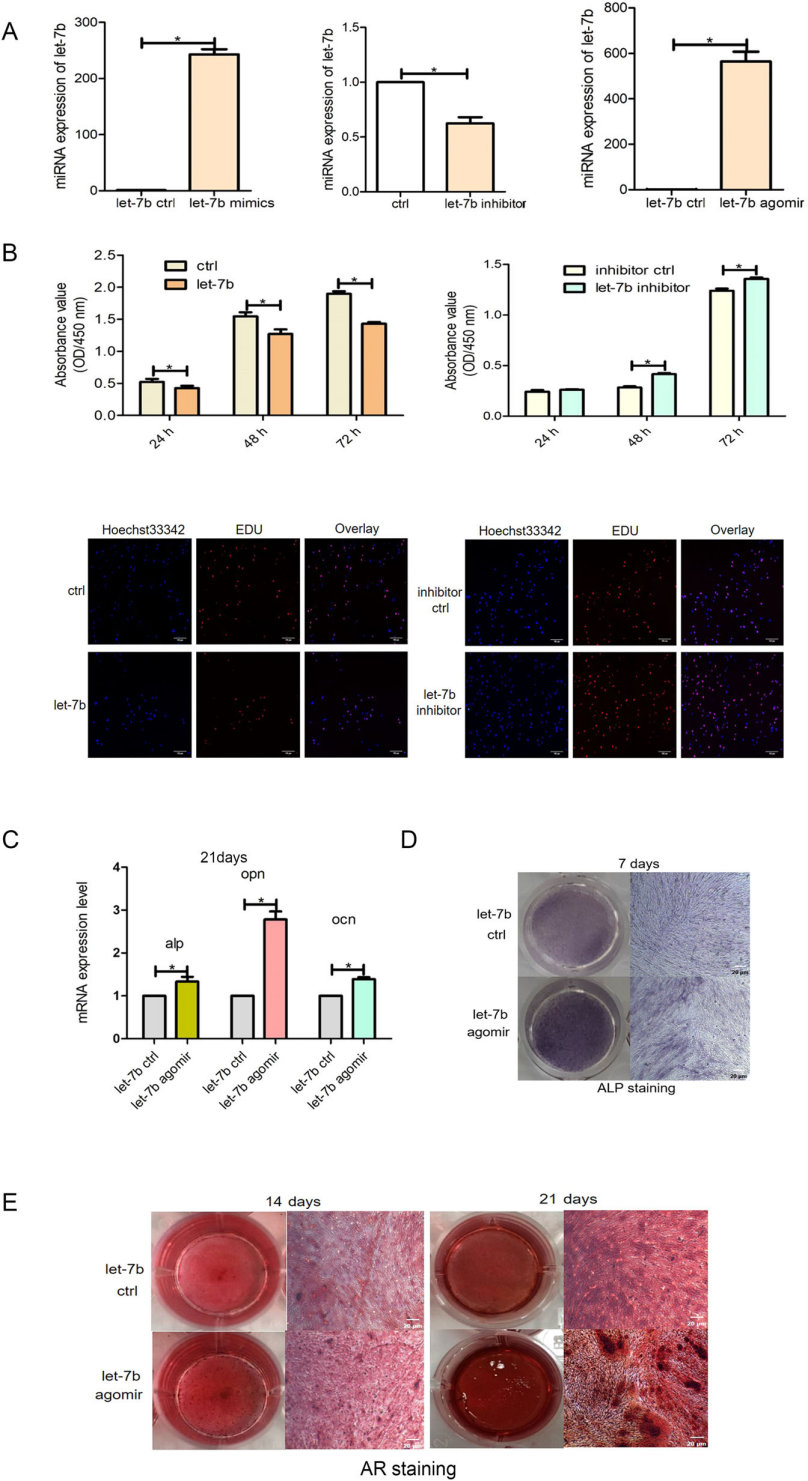
Effect of let-7b on the proliferation and differentiation ability of hDPSCs. **A** Detection of let-7b expression efficiency. **B** Effect of let-7b overexpression on the proliferative activity of hDPSCs. **C**–**E** PCR, ALP and alizarin red staining were used to detect osteogenic differentiation of hDPSCs after transfection with let-7b, respectively. Scar bar = 20 μm.

To investigate the role of let-7b in the biological characteristics of DPSCs, let-7b mimics, agomir, and inhibitor particles were transfected to enhance or knock down the expression of let-7b. qRT-PCR was performed to quantify the total mRNA level of let-7b (*P* < 0.05; Fig. 3A). The CCK-8 and EDU assays were then used to explore the effect of let-7b on cell proliferation. The cell proliferation of the let-7b mimic group was significantly reduced at three time points (*P* < 0.05; Fig. 3B), compared with that of the control group. The EDU assay shows a decrease in cell number after overexpression of let-7b. Conversely, the upregulation of let-7b mRNA expression showed that hDPSCs had enhanced odontoblastic and osteogenic differentiation capacities, as their total mRNA levels of osteogenesis-related genes, such as *ALP*, *OPN*, and *OCN*, also increased compared with the let-7b mimic control group (*P* < 0.05; Fig. 3C). More pronounced ALP and Alizarin red staining and an increased number of mineral nodules were also observed compared with that of the let-7b agomir control group (*P* < 0.05; Fig. 3D, E).

### Lin28 increases cell viability by targeting pre-let-7b

**Fig. 4:**
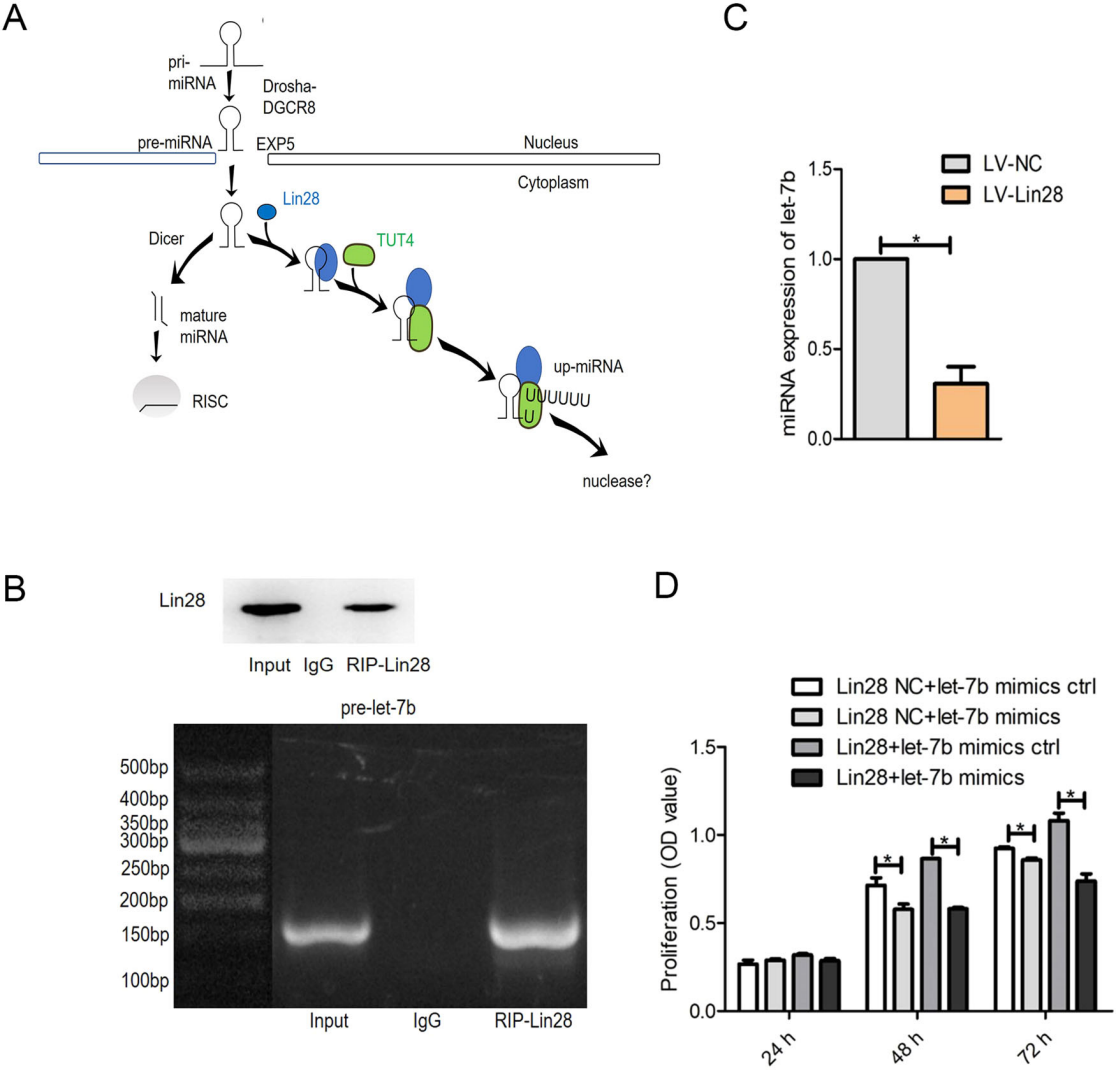
Evaluation of the targeting relationship between Lin28 and let-7b. **A** The mechanisms of Lin28 hinder let-7b maturation. **B** The RNA-binding protein Lin28 can enrich for the let-7b precursor pre-let-7b. **C** Expression levels of let-7b in hDPSCs overexpressing Lin28. **D** Overexpression of Lin28 in hDPSCs upregulates let-7b, and cell proliferation capacity is inhibited.

The upregulation of Lin28 and let-7b in hDPSCs prompted further research into their interactions. The biogenesis of miRNAs is summarized as follows. Following pre-miRNA transcription in the nucleus, it is processed by Drosha into a pre-miRNA with a hairpin structure, which is then transported out of the nucleus with the help of the Exportin-5 complex. This transported pre-miRNA is then cleaved into a mature miRNA by Dicer in the cytoplasm. It is then integrated into RNA silencing complexes (RISCs) that subsequently regulate gene expression through the complete or incomplete pairing of recognition sites, mainly located in the 3′-UTR of mRNA. Lin28 binds to pre-let-7b and recruits the terminal uridyltransferase (TUTase) Zcchc11 (TUTase4/TUT4), which adds an oligouracil tail to the 3′ end of pre-let-7 to block Dicer cleavage and inhibit the production of mature let-7b (Fig. 4A).

To determine whether Lin28 affects the biological properties of cells through let-7b, we performed RIP-Chip analysis to detect the interaction between Lin28 and let-7b. The specific binding combination of Lin28 to let-7b was visualized via RIP-PCR using immunoprecipitation obtained with IgG as a negative control. It was experimentally verified that Lin28 mRNA was significantly enriched in the RIP and input, and overall, these results showed that Lin28 could specifically bind to let-7b precursors and inhibit the maturation of let-7b (Fig. 4B).

As expected, qRT-PCR showed a significant reduction in *let-7b* levels in LV-Lin28-transfected hDPSCs compared with scrambled miRNA-transfected hDPSCs (*P* < 0.05; Fig. 4C). This shows that the increased expression of Lin28 reduces endogenous let-7b levels. At the same time, the CCK-8 assay revealed an inverse effect on cell viability between let-7b and Lin28, with Lin28 producing a positive effect and let-7b showing the opposite effect (*P* < 0.05; Fig. 4D). Specifically, let-7b mimics remarkably reversed the enhancement of cell proliferation induced by Lin28 expression (*P* < 0.05; Fig. 4D).

### let-7b inhibits cell proliferation by directly targeting IGF2BP2

**Fig. 5:**
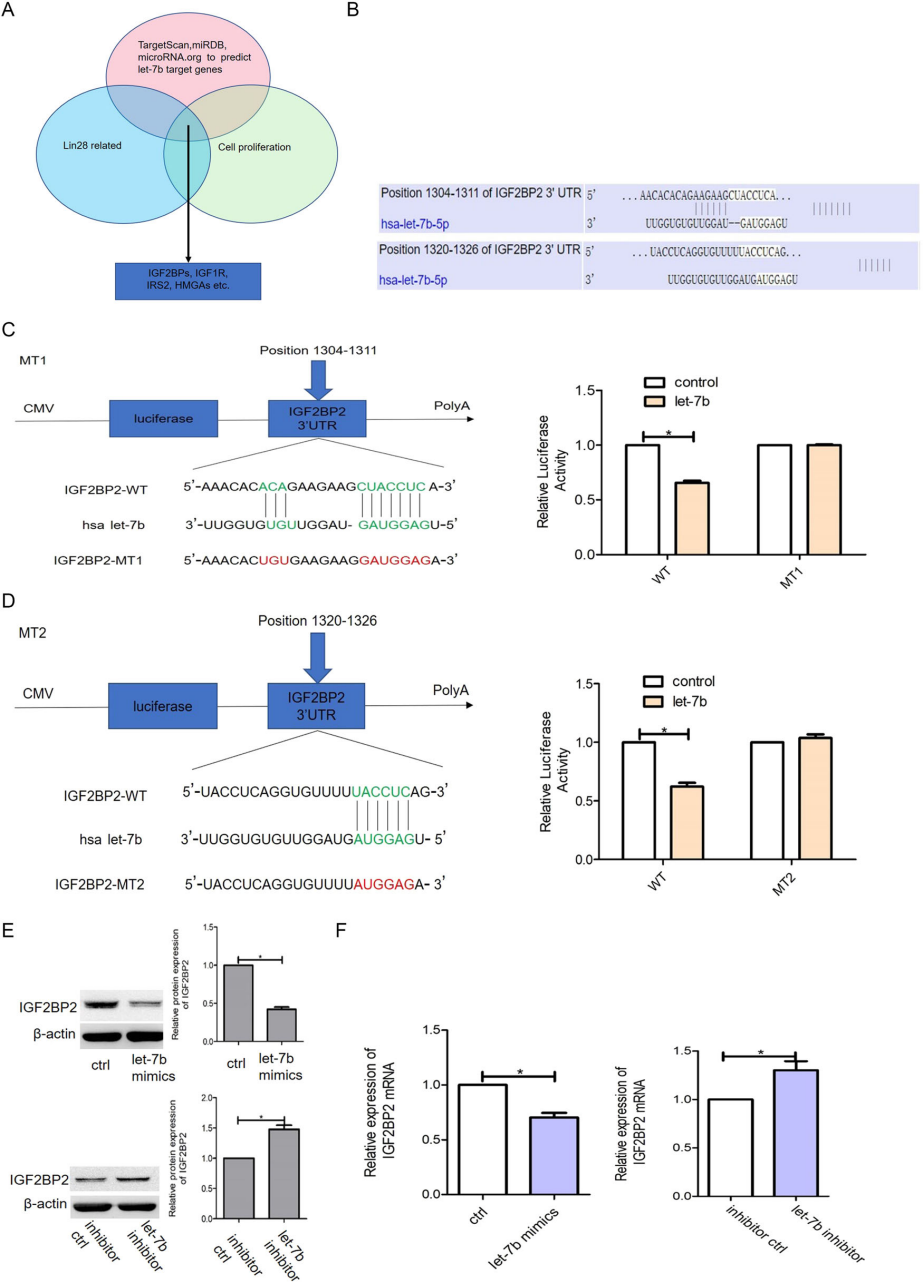
Evaluation of let-7b and IGF2BP2 targeting relationship. **A** Three bioinformatics software to predict the target mRNA of let-7b. **B** Software predicts the let-7b binding site for IGF2BP2 mRNA. **C**, **D** Dual luciferase assay was used to detect whether let-7b binds the 3’UTR region of IGF2BP2. Position 1304-1311 binding site of let-7b to the 3’UTR of IGF2BP2 and dual luciferase assay to detect whether let-7b and IGF2BP2 are targeted for binding (**C**). Position 1320-1326 binding site of let-7b to the 3’UTR of IGF2BP2 and dual luciferase assay to detect whether let-7b and IGF2BP2 are targeted for binding (**D**). **E**, **F** Western blotting and RT-PCR was used to detect the effect of let-7b overexpression and knockdown on IGF2BP2 expression.

Online prediction software (TargetScan 7.1, microRNA.org, and miRDB) was used to identify the targets of let-7b and IGF2BP2 was found to be one of them (Fig. 5A, B). Luciferase reporter assays were performed, to verify the interaction between let-7b and the 3ʹ-UTR fragment of IGF2BP2, in order to experimentally confirm whether let-7b directly targets IGF2BP2 in hDPSCs. The 3ʹ-UTR of IGF2BP2 is the binding site of let-7b, named the wild-type (IGF2BP2-WT). Two mutant (IGF2BP2-Mut) binding sites were then subcloned into the region downstream of the pmiRGLO dual-luciferase reporter vector. Co-transfections of either let-7b or let-7b mimics (as controls) and luciferase reporter constructs comprising WT or Mut 3ʹ-UTRs were then conducted. When HEK 293 T cells were transiently transfected with the WT-3ʹ-UTR-reporter, let-7b showed significantly decreased relative luciferase activity compared to cells transfected with let-7b mimics. However, the luciferase activity of the reporter carrying the Mut-3ʹ-UTRs was unaffected by co-transfection with let-7b (Fig. 5C, D). These results strongly indicate that let-7b can bind directly to the 3ʹ-UTR of IGF2BP2.

To determine whether let-7b expression affects endogenous IGF2BP2 expression, hDPSCs were transfected with let-7b, a let-7b inhibitor, or negative controls. The mRNA and protein expression of IGF2BP2 decreased in cells transfected with let-7b mimics and increased in cells transfected with the let-7b inhibitor (Fig. 5E, F). Overall, these results proved that let-7b regulated the expression of IGF2BP2 by directly targeting its 3ʹ-UTR.

### IGF2BP2 knockdown mimics the effects of let-7b overexpression

**Fig. 6:**
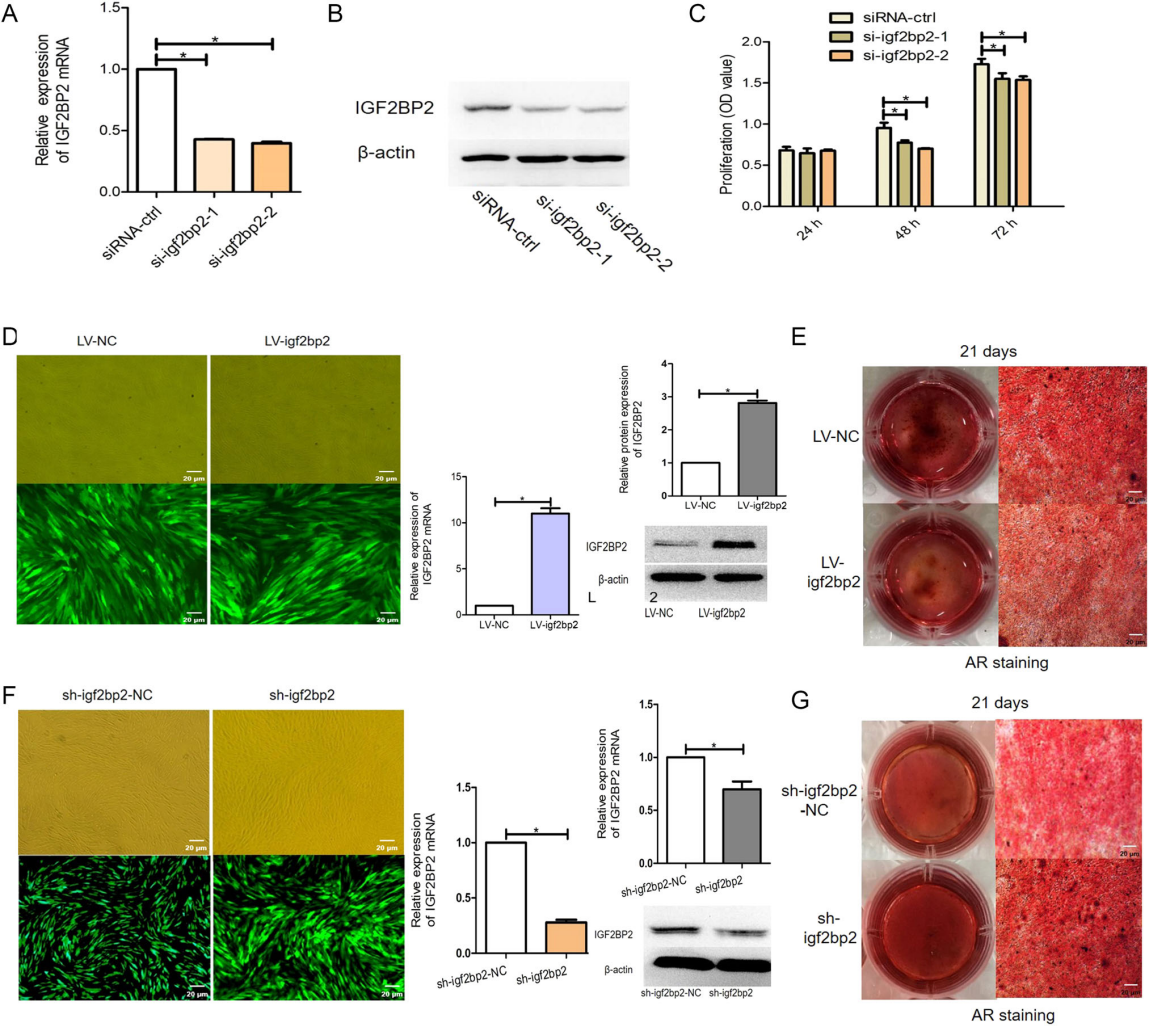
Effect of IGF2BP2 on the proliferation and differentiation ability of hDPSCs. **A**, **B** Detection of si-igf2bp2 silencing efficiency and screening of the best silencing efficiency sequence. **C** Effect of IGF2BP2 on the proliferative activity of hDPSCs. **D**, **E** Stable overexpression of IGF2BP2 and detection of its effect on the differentiation activity of hDPSCs. Overexpression efficiency of IGF2BP2 detected by microscopy, PCR and western blotting (**D**). Effect of elevated IGF2BP2 expression on dentin-oriented differentiation of pulp stem cells detected by alizarin red staining (**E**). **F**, **G** Stable knockdown of IGF2BP2 and detection of its effect on the differentiation activity of hDPSCs. Downregulation efficiency of IGF2BP2 detected by microscopy, PCR and western blotting (**F**). Effect of reduced IGF2BP2 expression on dentin-oriented differentiation of pulp stem cells detected by alizarin red staining (**G**). Scar bar = 20 μm.

For a more comprehensive understanding of the role of IGF2BP2 on hDPSCs, IGF2BP2-knockdown cells were transfected with siRNA, which interferes with IGF2BP2 expression. IGF2BP2 expression was reduced at both protein and mRNA levels (*P* < 0.05; Fig. 6A, B). Analyzing the absorbance values at 450 nm, an inverse relationship between cell differentiation and IGF2BP2 level was observed in the siRNA-IGF2BP2 group (*P* < 0.05; Fig. 6C), simulating the proliferative effect of let-7b.

To understand the effect of IGF2BP2 on the biological properties of cells, we successfully constructed an IGF2BP2 overexpression vector, obtaining stable overexpression cell lines via LV transfection. These cells had a significantly increased IGF2BP2 expression at the mRNA and protein levels (*P* < 0.05; Fig. 6D). Additionally, the number of mineralized nodules significantly increased in the LV-IGF2BP2 group (Fig. 6E), however the expression of both IGF2BP2 mRNA and protein decreased after shRNA-mediated post-transcriptional silencing, as shown by western blotting and qRT-PCR, respectively (*P* < 0.05; Fig. 6F). Compared with that in sh-IGF2BP2-NC, the intensity of Alizarin red staining and the formation of mineralized nodules in the sh-IGF2BP2 group increased (Fig. 6G). These results suggest that silencing IGF2BP2 inhibits the proliferation and promotes the differentiation of hDPSCs.

### Effect of Lin28/let-7b/IGF2BP2 on the proliferation and differentiation activity of hDPSCs

**Fig. 7:**
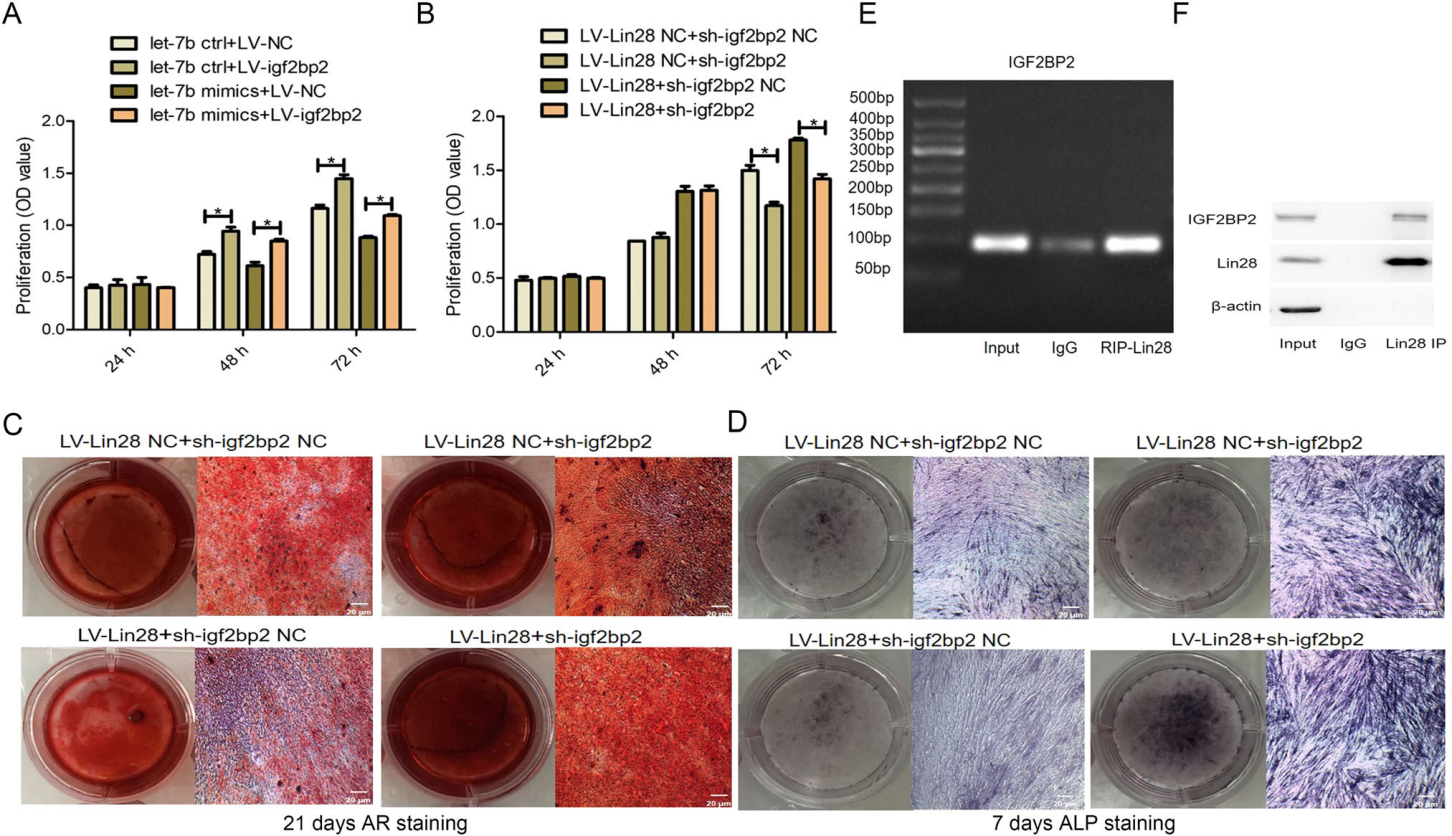
Detection of Lin28/let-7b/IGF2BP2 on the proliferation and differentiation ability of hDPSCs. **A** Overexpression of IGF2BP2 attenuates the inhibition of let-7b on hDPSCs. **B** Silencing of IGF2BP2 attenuates the promotion of Lin28 on hDPSCs. **C**, **D** Effect of overexpression and silencing of IGF2BP2 on the differentiation activity of hDPSCs overexpressing Lin28. **E**, **F** There is no targeting relationship between Lin28 and IGF2BP2. Scar bar = 20 μm.

To further explore the effect of regulating the Lin28/let-7b/IGF2BP2 axis on the biological characteristics of cells, we overexpressed IGF2BP2 along with let-7b and examined the restorative effect of IGF2BP2 on the let-7b-induced inhibition of hDPSC proliferation. The CCK-8 assay revealed that, compared to the let-7b ctrl+LV-NC group, the number of proliferating cells in the let-7b ctrl+LV-IGF2BP2 group was significantly increased (*P* < 0.05; Fig. 7A), indicating that the overexpression of IGF2BP2 could promote the proliferation of hDPSCs. Compared to the let-7b mimics+LV-NC group, IGF2BP2 overexpression reversed the let-7b-induced inhibition of hDPSC proliferation. Similarly, compared with the LV-Lin28 NC+sh-IGF2BP2 NC group, the CCK-8 assay of LV-Lin28 NC+sh-IGF2BP2 showed that silencing IGF2BP2 reduced the cell proliferative effect of Lin28. Compared with LV-Lin28+sh-IGF2BP2 NC, the LV-Lin28+sh-IGF2BP2 group showed that silencing IGF2BP2 inhibits the promotive effect of Lin28 on hDPSC proliferation (*P* < 0.05; Fig. 7B).

The osteogenic differentiation capacity of hDPSCs was also evaluated via Alizarin Red S and ALP staining after 14 d of osteogenic induction. Mineralized matrix deposition was increased in the LV-Lin28+sh-IGF2BP2 group compared to that in the LV-Lin28+sh-IGF2BP2 NC group, as was the intensity of ALP staining (Fig. 7C, D). These results show that IGF2BP2 knockdown attenuated the effect of Lin28 on promoting hDPSC proliferation and inhibiting odontoblastic and osteogenic differentiation.

Next, we used RIP assays to analyze the association of Lin28 with IGF2BP2 mRNA and explore the relationship between Lin28 and IGF2BP2. Both the control IgG and RIP-Lin28 groups were enriched in IGF2BP2 mRNA, indicating that Lin28 could not bind to IGF2BP2 mRNA (Fig. 7E). In addition, we performed immunoprecipitation to detect the direct binding between the Lin28 and IGF2BP2 proteins. The results showed that Lin28-specific antibodies could pull down IGF2BP2 without nonspecific binding (Fig. 7F).

## Discussion

This study analyzed the changes in the proliferation and osteogenesis-related mRNA and protein expression of hDPSCs after transfection with Lin28-shRNA, let-7b-miRNA, and IGF2BP2-siRNA LV vectors, suggesting that the biological characteristics of the hDPSCs were affected. Gronthos discovered hDPSCs for the first time in 2000, which are highly proliferative cells and have the ability to form cell clones, and can differentiate into various cells such as osteoblasts, chondrocytes, adipocytes, and neuronal cells under the conditions of induction. Compared with other stem cells such as mesenchymal stem cells, hDPSCs are easy to obtain, have low immunogenicity, and can form dispersed and high-density calcified nodules and dentin cell-like cells when induced in vitro, and can form dentin/pulp-like complexes [27]. The results revealed the existence of certain regulatory mechanisms of the Lin28, let-7b, and IGF2BP2 genes in the osteogenic differentiation of hDPSCs. Dentin salivary phosphoprotein (DSPP), as an extracellular matrix protein closely related to tooth development, contains dentin phosphoprotein (DPP) and dentin salivary phosphoprotein (DSP). DPP has a nucleating role in the biomineralization of dentin and also regulates the morphology and growth rate of mineralized crystals, which is a key regulator of mineralized tissue stabilization. DSP is a glycophorin, which is a key regulator of mineralized tissue stability. DSPP is a gene complex of DPP and DSP and is considered the major dentin-specific protein. DSPP is a specific marker that distinguishes odontoblast cells from other cells [28]. Dentin matrix protein 1 (DMP1), containing an arginine-glycine-aspartate motif, is non-collagenous protein. During osteoblast differentiation and maturation, DMP1 protein is phosphorylated and then translocated to the extracellular matrix to assist in the formation of dentin matrix. Therefore, DMP1 plays an important regulatory function in bone and dentin mineralization [29]. In the present study we did not perform tests for these two indicators, but only for ALP staining and alizarin red staining to assess the ability of hDPSCs to differentiate into odontoblast, which is a drawback of the article.

Biological function analysis revealed that the effects of Lin28 and IGF2BP2 were consistent, promoting cell proliferation while inhibiting differentiation. At the same time, let-7b mRNA can directly bind to Lin28 mRNA, producing the opposite effect. The interactions between these three genes were validated via IP. The RIP analysis suggests that Lin28 could downregulate the expression of mature let-7*b* mRNA by binding to its precursor. We next checked if let-7b identified IGF2BP2 as a novel target and bound directly to its 3′-UTR. Our in vitro results showed that let-7b negatively regulated IGFBP2 expression and influenced hDPSC proliferation and differentiation. Overall, these experiments illustrated that the non-targeted binding of Lin28 to IGF2BP2 mRNA affects the biological properties of hDPSCs by modulating let-7b expression.

IGF2BP2 has been identified as a promoter of diabetes mellitus and an oncogene that promotes several types of tumors [19, 30, 31]; however, its role in hDPSC proliferation and differentiation remains elusive. We studied the possible interaction between Lin28 and IGF2BP2 in hDPSCs, revealing how Lin28 suppresses IGF2BP2 expression by downregulating let-7b. Based on our findings, we propose a working model of how Lin28 and IGF2BP2 interact to modulate multiple cellular functions and suppress osteogenic differentiation by inhibiting the maturation of pre-let-7b and downregulating let-7b mRNA expression. Identifying molecules that simultaneously regulate bone cell differentiation and proliferation could be a direction of future research into bone regeneration and reconstruction.

Lin28, a highly conserved RNA-binding protein first identified in *Cryptobacterium hidradenum* in 2000 [32], is a core stem cell factor that regulates the biogenesis of embryonic stem cells and various cancer cell lines in a pleiotropic manner [26], reprograms translation to promote the progression of various metabolic diseases and cancers, and plays a regulatory role in the transcriptional regulation of let-7 [16]. Let-7b is a member of the let-7 family, a 13-member family of highly conserved microRNAs widely found in living organisms. It has been shown that the overexpression of let-7 in hDPSCs inhibits cell proliferation and blocks the DNA synthesis (S) and cell growth (G2)/mitotic (M) phases of the cell cycle [13], which is consistent with the experimental results of the present study. A growing body of data suggests that let-7 miRNAs are tumor suppressors and cell cycle regulators [33–39]. They induce an orderly entry into the terminal differentiation phase of cell development and participate in forming the miRNA-induced silencing complex RISC, which binds to the 3′-UTR of target genes through base complementary pairing to mediate the recognition and downregulation of mRNA [40]. Together with Lin28, let-7 maintains the homeostasis of stem cell function [41], acting as a heterochronous gene to regulate the developmental timing of stem cells. In the present study, we found that the expression of Lin28 regulates the maturation and expression level of let-7b, which synergistically affects the proliferation and differentiation of hDPSCs.

IGF2BP2, a member of the insulin-like growth factor 2 mRNA-binding protein family, is related to type 2 diabetes-related genes [42, 43] reported that locus variants in IGF2BP2, such as genetic polymorphisms, inhibited the ability of insulin secretion in patients with type 2 diabetes during the disease progression phase in Dutch and German populations. [23] observed a significant improvement in glucose tolerance and insulin sensitivity in IGF2BP2-knockout mice. However, recent studies have shown that IGF2BP2 is also an m6A reader. It contains four K-homologous structural domains and two RNA recognition motifs that can affect the post-transcriptional regulation of various genes [44]. Through communication with miRNAs, lncRNAs, mRNAs, and other m6A-related genes [20], it can regulate cellular metabolism and influence biological processes. IGFBP2 is significantly associated with poor prognosis in some cancers and metabolic diseases [45] and could promote angiogenesis, tumorigenesis, and metastasis. Targeting the regulatory mechanism of IGF2BP2 may promote the development of new therapeutic strategies for diabetes [46], fatty liver [22], and pancreatic [47], liver [31], breast [13, 48], and oral cancers [21]. [31] showed that IGF2BP2 mediates the nuclear localization of p65 and promotes hepatocellular carcinoma (HCC) metastasis through the nuclear factor kappa-light-chain-enhancer of activated B cells (NF-κB)/ Zinc finger E-box binding homeobox 1 (ZEB1) axis, an independent indicator of HCC. Thus, targeting IGF2BP2 could be a promising therapeutic strategy for hepatocellular carcinoma.

Zhang et al. [49] found that IGF2BP2 targets and stabilizes serum response factor (*SRF*) mRNA, regulates osteogenic differentiation (OGD), and can be a potential marker and therapeutic target for osteoporosis and OGD. Although the role of IGF2BP2 in cancer has been extensively studied, the mechanism by which it regulates cell proliferation and differentiation remains unclear. Our preliminary study [13] confirmed the novel regulatory mechanism involving the let-7/IGF2BP2 axis in hDPSCs. Let-7 directly targets IGF2BP2, which controls the expression of proliferation-related genes and accelerates the S and G2/M phases in hDPSCs. Therefore, we proposed a method to regulate the Lin28/let-7/IGF2BP2 axis and improve the biological characteristics of stem cells.

Our study showed that IGF2BP2 overexpression promoted the proliferation and inhibited the differentiation of hDPSCs in vitro, which is consistent with the effect of Lin28 overexpression. To confirm the regulatory mechanism of the Lin28/let-7b/IGF2BP2 axis in cementoblastic and osteogenic differentiation, LV transfection was used to upregulate the expression and siRNA and shRNA to downregulate the expression of target genes. RIP sequencing and luciferase assays identified target RNAs interacting with IGF2B2 and multiple binding sites in the IGF2BP2 3′-UTR. The results showed that let-7b had the opposite effect on Lin28 and IGF2BP2. Lin28 enhances the expression of pro-proliferative genes by promoting their translation and provides a positive feedback mechanism for changes in the biological characteristics of hDPSCs. Downregulation of the Lin28/let-7b/IGF2BP2 axis can kick-start odontoblasts, providing new insights into the roles of Lin28 and let-7 in the pluripotent reconfiguration of cells. Although this study showed that the activation of the Lin28/IGF2BP2 pathway has a promising role in pulpal periodontal tissue regeneration and bone tissue engineering, in vitro experiments cannot represent the true role of this pathway in vivo. Hence, further in vivo experiments and clinical trials are needed to verify the results of the present study.

In conclusion, we explored the role of Lin28 in osteogenic differentiation of hDPSCs. This study provides new evidence that let-7b can directly target the 3’UTR of IGF2BP2 mRNA, which regulates the expression of differentiation-related genes. Our results also provide a new theoretical basis for Lin28 to regulate damage repair in tissues. Subsequently, the mechanism by which the Lin28/let-7/IGF2BP2 axis achieves optimal proliferation while initiating the differentiation of hDPSCs to osteoblasts needs to be further investigated.

## Data Availability

The authors confirm that the data supporting the findings of this study are available within the article and its supplementary materials.
